# Production of d-Tagatose by Whole-Cell Conversion of Recombinant *Bacillus subtilis* in the Absence of Antibiotics

**DOI:** 10.3390/biology10121343

**Published:** 2021-12-16

**Authors:** Xian Zhang, Ruiqi Lu, Qiang Wang, Mengkai Hu, Zhiyue Li, Meijuan Xu, Taowei Yang, Rongzhen Zhang, Zhiming Rao

**Affiliations:** The Key Laboratory of Industrial Biotechnology of Ministry of Education, School of Biotechnology, Jiangnan University, Wuxi 214122, China; zx@jiangnan.edu.cn (X.Z.); 6200208033@stu.jiangnan.edu.cn (R.L.); 6200201091@stu.jiangnan.edu.cn (Q.W.); 7180201039@stu.jiangnan.edu.cn (M.H.); 6180202003@stu.jiangnan.edu.cn (Z.L.); xumeijuan@jiangnan.edu.cn (M.X.); yangtw@jiangnan.edu.cn (T.Y.)

**Keywords:** *Bacillus subtilis*, d-alanine racemase, d-tagatose, arabinose isomerase, β-galactosidase, food-grade, whole-cell bioconversion

## Abstract

**Simple Summary:**

d-tagatose is a valuable monosaccharide in the food industry produced from lactose by β-galactosidase and arabinose isomerase. To improve its production safety, d-alanine-deficient heterologous gene expression systems were constructed without antibiotics. The integrated expression and co-expression plasmids were used in different systems, also exploiting the need for d-alanine during cellular metabolism. The integration of the β-galactosidase gene in recombinant is uniquely innovative and promising, applying common knockout techniques to the expression of target genes and the production of high-value products.

**Abstract:**

d-tagatose is a popular functional monosaccharide produced from lactose by β-galactosidase and arabinose isomerase. In this study, two d-alanine-deficient heterologous gene expression systems were constructed, *B. subtilis* 168 D1 and *B. subtilis* 168 D2, using overlapping extension PCR and the CRE/*loxP* system. The *lacZ* gene for β-galactosidase was integrated into a specific locus of the chassis *B. subtilis* 168 D2. A mutually complementary plasmid pMA5 with the alanine racemase gene *alrA* attached to it was constructed and used to assemble recombinant plasmids overexpressing β-galactosidase and arabinose isomerase. Afterward, an integrated recombinant was constructed by the plasmid expressing the arabinose isomerase gene *araA* of *E. coli* transform-competent *B. subtilis* 168 D2 cells. The co-expressing plasmids were introduced into alanine racemase knockout *B. subtilis* 168 D1. Whole-cell bioconversion was performed using the integrated recombinant with a maximum yield of 96.8 g/L d-tagatose from 500 g/L lactose, and the highest molar conversions were 57.2%. *B. subtilis* 168 D1/pMA5-*alrA*-*araA-lacZ* is capable of single-cell one-step production of d-tagatose. This study provides a new approach to the production of functional sugars.

## 1. Introduction

d-tagatose is a popular monosaccharide with sweetness similar to sucrose but has 30% fewer calories, possesses probiotic properties that improve intestinal flora, and has desirable coloring properties. Therefore, d-tagatose is an optimal alternative sweetener and is widely used in healthy foods [1,2]. d-tagatose has been recognized as safe (GRAS) by the US Food and Drug Administration (https://www.fda.gov/, accessed on 1 December 2001) [3] and was approved as a safe food in 2004 by the Joint Expert Committee on Food Additives of the World Health Organization. The global artificial sweetener market, estimated to be $3.2 billion in 2016 [4], is expected to expand, and so safe and high-yield production of d-tagatose has become a pressing problem.

“Integrative expression” is the insertion of an exogenous gene into a position on a chromosome, where it stably replicates with the host chromosome. After sequencing the whole genome of *Bacillus subtilis*, the integration vector technique expanded, with *B. subtilis* becoming the most widely used microorganism in industry. Nevertheless, integrated plasmids used to insert exogenous genes can carry resistance genes, restricting their use in the food industry. In this study, an antibiotic-free integrated expression system was designed using a CRE/*loxP*-specific recombination system and d-alanine racemase activity.

Arabinose isomerase has the ability to catalyze the synthesis of d-tagatose from galactose [5]. Most arabinose isomerases are from mesophilic, thermophilic, and hyperthermophilic bacteria (Table S1). The conversion of galactose to d-tagatose by arabinose isomerase is costly because of problems, such as unfavorable kinetics [5], low thermal stability, and low equilibrium constant (the ratio of galactose to d-tagatose is about 7:3) [4]. Less costly raw materials have been used to hydrolyze lactose and simultaneously synthesize d-tagatose using arabinose isomerase [6]. β-galactosidase, or lactase, can hydrolyze or transglycosylation of galactose or galactooligosaccharides, respectively [7]. β-galactosidase derived from various sources has different ratios of hydrolytic activity and transglycosidase activity. β-galactosidase derived from *E. coli* possesses high hydrolytic activity, while those derived from *B. circulans*, *Bifidobacterium bifidum*, or *Aspergillus oryzae* have intense transglycosidic activity [8,9,10].

The key enzymes β-galactosidase and arabinose isomerase derived from *E. coli* are used for d-tagatose synthesis [11]. Most production still uses *E. coli*, which may result in the release of enterotoxins that compromise food safety during synthesis of some food additives [6,12]. Multi-copy plasmid expression systems are usually constructed to efficiently express target proteins, and antibiotic resistance genes have been used as selection pressure to maintain the genetic stability of the plasmid in host cells. However, the use of antibiotics in the food industry is not allowed. As a GRAS strain often used for fermentation, *B. subtilis* has increasingly become a food-grade host used to synthesize enzymes, fine chemicals, and food additives [13,14].

In this study, a d-alanine-deficient recombinant strain of bacteria that integrates the β-galactosidase *lacZ* gene from *E. coli* and alanine racemase knockout chassis was constructed. A plasmid expressing alanine racemase, pMA5-*alrA*, was also constructed and used as a basis for expressing β-galactosidase and arabinose isomerase. These recombinant plasmids were transformed into an expression system to obtain co-expressing recombinant strains that produce d-tagatose. The optimal temperature, pH, (Mn^2+^), cell permeabilization, and density were investigated for their effects on whole-cell conversion. Production of d-tagatose under optimal transformation conditions provides new insights into the biological production of a new generation of reduced sugars used as food ingredients.

## 2. Materials and Methods

### 2.1. Bacterial Strains, Plasmids, and Culture Conditions

The bacterial strains and plasmids used in this study are listed in Table S2. The basic medium used for bacterial culture was Luria Bertani (LB) (peptone 10 g/L, NaCl 10 g/L, yeast powder 5 g/L). The medium used for fermentation was Terrific Broth (TB) (yeast powder 24 g/L, peptone 12 g/L, Na_2_HPO_4_•3H_2_O 16.4 g/L, NaH_2_PO_4_ 2.3 g/L, glycine 7.5 g/L, glycerol 5 g/L) with 2% agar used as a solid medium. When used, antibiotics or d-alanine were added: ampicillin (100 µg/mL), kanamycin (50 µg/mL), bleomycin (30 µg/mL), and d-alanine (100 µg/mL). A single colony was inoculated into 10 mL of LB liquid medium and cultured for 12 h, followed by inoculation into 50 mL of TB fermentation medium at 1% and cultured for an additional 24 h. All strains were cultured at 37 °C in a shaker incubator at 180 rpm.

### 2.2. Construction of Expression Systems

The upstream and downstream 800 bp sequences of the gene *alrA* (alanine racemase) in *B. subtilis* 168 and the *E. coli*-derived β-galactosidase gene *lacZ* were amplified by PCR. From the p7Z6 plasmid using primers designed to amplify the lox71-zeo^r^-lox66 cassette, the long fragments were fused using overlapping and extension PCR, and then introduced into *B. subtilis* 168 separately by a chemical method. The thermosensitive pTSC plasmid was introduced into the recombinant strain to eliminate the introduced bleomycin resistance gene. The pTSC plasmid was eliminated by incubating the bacterial strains on plates supplemented with d-alanine at 51 °C for 48 h. Colonies were seeded onto separate d-alanine plates containing either no antibiotic or bleomycin and kanamycin to select for the target recombinant strain. Similarly, a simple alanine racemase knockout strain was constructed in the same way after extended-fragment synthesis, which did not include the *lacZ* gene.

The recombinant strains were validated by PCR using upstream and downstream primers from the upstream and downstream homology arms, with the original *B. subtilis* 168 as a control group, and construction of the recombinant strains was judged from electrophoretic bands in agarose gels.

Primers were designed to amplify *alrA* in the *Bacillus subtilis* 168 genome and to amplify a pMA5 plasmid fragment without the kanamycin and bleomycin antibiotic genes by reverse amplification. After purification and digestion with *Dpn*I enzyme, the pMA5 plasmid fragment was ligated to the purified *alrA* fragment. The ligation product was then introduced into d-alanine-deficient chassis cells in culture without the addition of antibiotics.

### 2.3. Construction of Recombinant Vectors 

Primers were designed to amplify the β-galactosidase (*lacZ*) and arabinose isomerase (*araA*) genes of *E. coli* K-12. The two target genes were inserted between the restriction sites *Nde*I and *Mlu*I in the pMA5-*alrA* vector. The products were transformed into the competent alanine racemase knockout cells to obtain food-grade recombinant plasmids expressing the *araA* and *lacZ* genes. The plasmid expressing arabinose isomerase integrated with β-galactosidase was introduced into chassis cells to obtain integrated co-expression recombinant strains. 

Primers were designed to amplify the *Hpa*II promoter and the arabinose isomerase *araA* fragment on the recombinant plasmid and inserted between the restriction sites *Kpn*I and *Hind*III of the recombinant plasmid expressing β-galactosidase to obtain the co-expression vector. The plasmids over-expressing both enzyme genes were transformed to the alanine racemase knockout strain to obtain free co-expression recombinant strains.

### 2.4. Construction of Expression Systems 

To test the stable heritability of the plasmid in the recombinant bacterium, the recombinant *Bacillus subtilis* was inoculated every 20 generations in LB medium containing 100 μg/mL d-alanine or no d-alanine in another identical medium and spread on LB plates containing d-alanine at multiples of 10^−4^, respectively, and incubated at 37 °C for 12 h. The individual colonies grown were spotted in LB plates with or without d-alanine and incubated for 12 h at 37 °C. The growth on the plates was observed to obtain the number of colonies grown on the different plates for a total of 100 generations. In order to compare the stability of food-safe recombinant strains with that of conventionally engineered recombinant strains, *B. stubtilis* 168 carrying the pMA5 plasmid was tested for plasmid genetic stability in the same way.

### 2.5. Enzyme Activity Assay 

The enzyme activities of β-galactosidase and arabinose isomerase were assessed by the amount of glucose or d-tagatose, respectively, produced in a whole-cell reaction. The 5 mL reaction mixture contained 100 g/L lactose substrate, 2 mL whole cells, 0.2 mol/L pH 8 buffer, 0.5 mol/L Mn^2+^, and was carried out at 50 °C, for 30 min prior to termination of the reaction (100 °C water bath for 10 min). One unit of β-galactosidase activity is defined as the amount of enzyme that catalyzes the production of 1 μmol of glucose per minute. One unit of arabinose racemase activity is defined as the amount of enzyme that catalyzes the production of 1 μmol of d-tagatose per minute. HPLC was used to detect the compounds in the reaction solution. HPLC used a Carbomix-Ca-NP chromatographic column and a refractive index detector, eluted with ultrapure water at a flow rate of 0.6 mL/min at 80 °C.

### 2.6. d-tagatose Production by Whole-Cell Bioconversion

In order to optimize the operating conditions, the effects of temperature and pH on the whole-cell activity of the recombinant strains were evaluated in the range 30–37 °C and pH 5.0–10.0, respectively. At the same time, the effect of 0–1 mol/L Mn^2+^ on whole-cell transformation was investigated. As *B. subtilis* is a Gram-positive bacterium with high levels of peptidoglycan in its cell wall, different concentrations of Triton X-100 nonionic surfactant, ranging from 0 to 1%, were used. Different concentrations of whole cells (OD = 10–60 at 600 nm wavelength) were tested.

Lactose was converted to d-tagatose by the recombinant whole cells under the optimum reaction conditions tested above. The volume of the recombinant cells was one liter. The substrate concentrations of lactose ranged from 100 to 500 g/L, and a 1 mL reaction sample was collected and centrifuged every 10 h to obtain the supernatant. The concentrations of the ingredients were measured by HPLC; 4 mol/L HCl and NaOH solutions were used to control pH during the process.

## 3. Results

### 3.1. Construction of a d-alanine-deficient Plasmid Expression System

**Figure 1:**
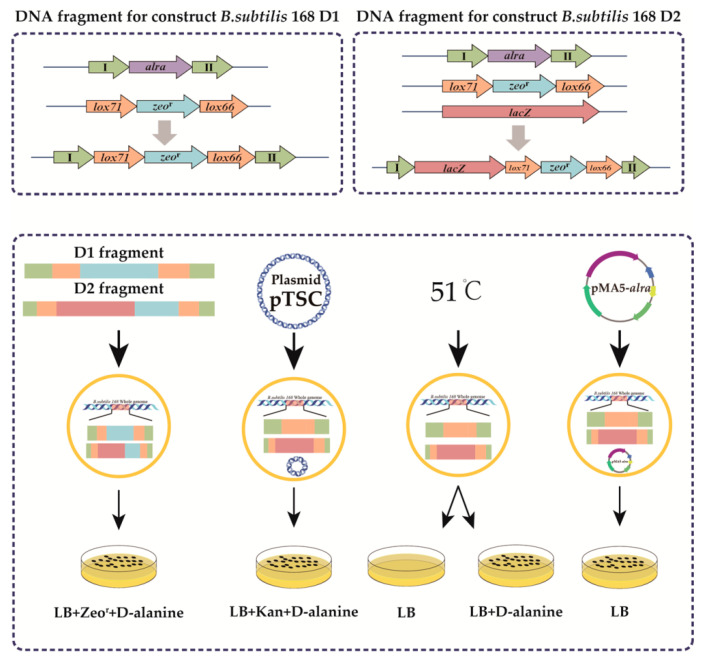
Strategy for the construction and genetic validation of food-grade host recombinant strains. Construction of a recombinant DNA fragment of the alanine racemase gene knocked out in *B. subtilis* 168. The upper and lower 800 bp fragments of the alanine racemase gene from the *B. subtilis* 168 genome and the lox71-zeo^r^-lox66 cassette amplified from the plasmid p7Z6 were fused into a recombinant DNA fragment of approximately 2.2 kb. Construction of a recombinant DNA fragment integrated the gene *lacZ* and knocked out the alanine racemase gene in *B. subtilis* 168. The upper and lower 800 bp fragments of the alanine racemase gene amplified from the *B. subtilis* 168 genomes, the *lacZ* gene amplified from *E. coli* K12, and the lox cassette from the plasmid p7Z6 were fused into a recombinant DNA fragment of approximately 5.2 kb.

The required homologous arm, the *E. coli*-derived β-galactosidase gene *lacZ*, and the lox71-zeo^r^-lox66 cassette in the p7Z6 plasmid were successfully amplified. The homologous arm and the lox71-zeo^r^-lox66 cassette were joined into a fragment of about 2 kb, amplified, and transformed *B. subtilis* 168 to obtain recombinant *B. subtilis* 168 D2P1. The homologous arm, the *lacZ* gene, and the lox71-zeo^r^-lox66 cassette were fused and amplified to fragments of 5 kb to obtain recombinant *B. subtilis* 168 D1P1. The thermosensitive pTSC plasmid was introduced into the two d-alanine racemase knockout hosts to eliminate the introduced bleomycin resistance gene, yielding *B. subtilis* 168 D1P2 and *B. subtilis* 168 D2P2. The pTSC plasmids were eliminated by incubating the -P2 strains on plates supplemented with d-alanine at 51 °C for 48 h. Colonies were seeded onto separate d-alanine plates containing either no antibiotic or bleomycin and kanamycin. Strains that did not grow on plates containing d-alanine and bleomycin and kanamycin but did grow on d-alanine plates containing no antibiotic were identified as recombinant *B. subtilis* lacking d-alanine and constituted the first step in validating the recombinant construct (Figure 1).

To further confirm that the expressed fragment of the gene *lacZ* was inserted into the alanine racemase *alrA*-specific locus of *B. subtilis*, a d-alanine-deficient recombinant strain was constructed by PCR amplification using the whole genome of the original *B. subtilis* as a template, with upstream primers from the upstream homology arm and downstream primers from the downstream homology arm.

The recombinant strain had a fragment of approximately 5 kbp, whereas the control only had a fragment of approximately 3 kbp. An approximately 1 kbp fragment of the alanine racemase gene (*alrA*) was successfully inserted into *B. subtilis*. Afterward, we sequenced the DNA of the integrated fragment and performed sequence alignment, again with the correct results.

### 3.2. Construction of Recombinant Strains, Expression, and Enzyme Activity

**Figure 2:**
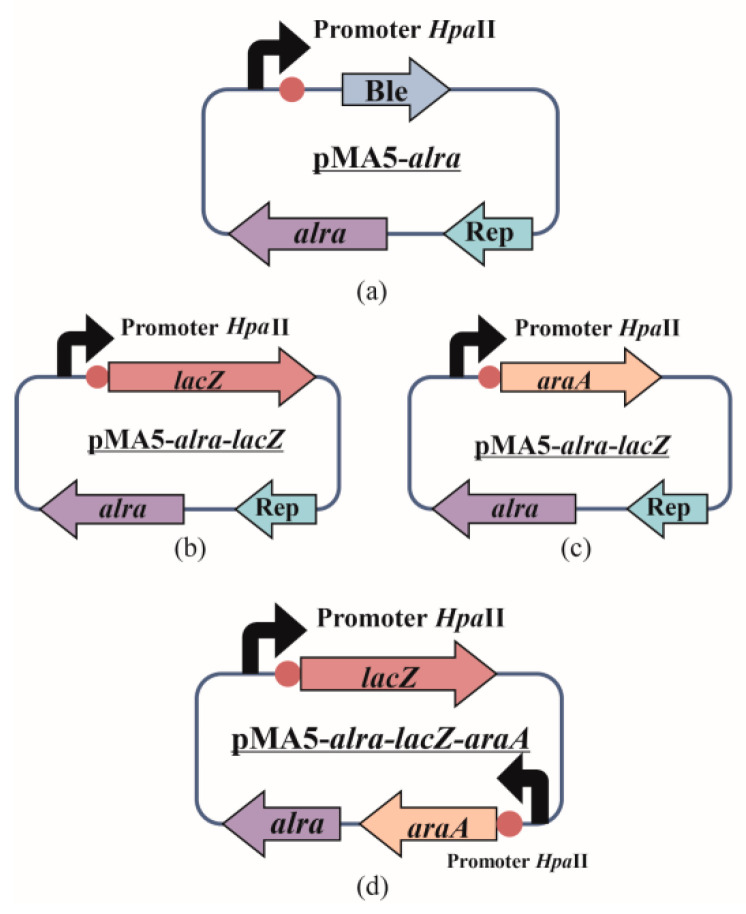
Strategy of the construction of food-grade expressing plasmids. (**a**) Construction of a plasmid pMA5-*alrA* expressing alanine racemase. Kanamycin and bleomycin were replaced by the enzyme alanine racemase *alrA* derived from *B. subtilis* 168 to construct expression plasmid pMA5-*alrA* based on plasmid pMA5. (**b**,**c**) The β-galactosidase and arabinose isomerase genes from *E. coli* K-12 were inserted between the multiple cloning sites *Nde*I and *Mlu*I of the constructed plasmid pMA5-*alrA* to separately construct plasmids pMA5-*alrA*-*lacZ* and pMA5-*alrA*-*araA*. (**d**) The arabinose isomerase gene was inserted between the *Kpn*I and *Hind*III sites from the constructed plasmids pMA5-*alrA*-*lacZ* to construct the co-expressing plasmid pMA5-*alrA*-*lacZ*-*araA*.

These genes (*lacZ* and *araA*) were amplified from the *E. coli* genome and were successfully amplified and separately inserted into plasmid pMA5-*alrA* to obtain the vectors pMA5-*alrA*-*lacZ* and pMA5-*alrA*-*araA*, respectively. The *B. subtilis* 168 D2/pMA5-*alrA*-*araA* was obtained by introducing the constructed plasmid pMA5-*alrA*-*araA* into the *B. subtilis* 168 D2. This recombinant plasmid was then used to successfully amplify the expression element *Hpa*II-*araA*, which was inserted into the recombinant plasmid pMA5-*alrA*-*lacZ* and introduced into the alanine-deficient strain *B. subtilis 168* D1 to obtain *B. subtilis 168* D1/pMA5-*alrA-lacZ-araA* (Figure 2).

**Figure 3:**
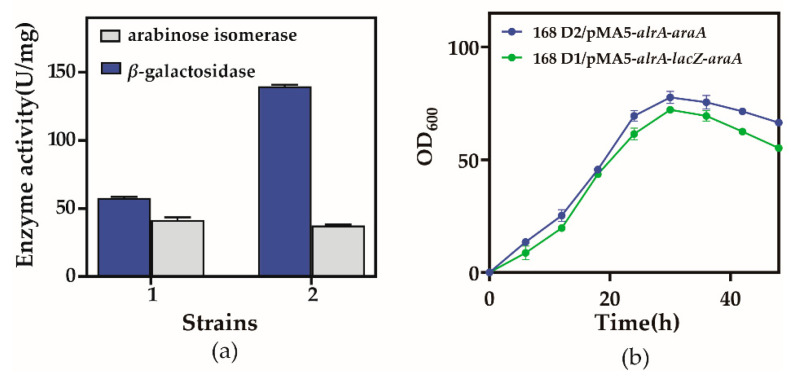
β-galactosidase and arabinose isomerase enzyme activity assay results and cell growth curves of the recombinant strains. (**a**) The 5 mL reaction mixture contained 100 g/L lactose substrate, 2 mL of whole cells, 0.2 mol/L pH 8 buffer, 0.5 mol/L Mn^2+^, and was carried out at 50 °C for 30 min prior to termination of the reaction (100 °C water bath for 10 min). 1: *B. subtilis* 168 D2/pMA5-*alrA*-*araA*; 2: *B. subtilis* 168 D1/pMA5-*alrA*-*lacZ*-*araA*. (**b**) Samples were taken every 6 h during culture at 40 °C, 160 r/min. Saline was used as a blank control for zeroing, and the optical density (OD) at a wavelength of 600 nm of a 20-fold dilution of the bacterial solution was measured. Results are expressed as mean ± SD from three independent experiments.

Recombinant colonies were inoculated into 10 mL of LB medium for 12 h, followed by inoculation into 100 mL of TB medium at 1% for 24 h. Bacteria were collected and lysed to obtain supernatant and pellet, and the supernatant contained expressed β-galactosidase and arabinose isomerase. *B. subtilis* 168 D2/pMA5-*alrA*-*araA* possessed enzymatic activities of 57.66 and 40.87 U/mg for β-galactosidase and arabinose isomerase, respectively, and in *B. subtilis* 168 D1/pMA5-*alrA*-*lacZ*-*araA*, the activities were 139.97 and 37.65 U/mg, respectively, with a significant advantage conferred by integration of *lacZ* (Figure 3a). The recombinant strain was seeded onto a fresh Luria Bertani (LB) medium solid plate, from which single colonies were selected and inoculated into 10 mL of LB medium in a shaker set to 37 °C for 12 h, diluted to 1% in 100 mL of LB medium at 40 °C in a shaker at 160 r/min, and sampled every 6 h. Saline was used as a blank control to adjust the zero level for measurement of the absorbance at the 600 nm wavelength of the 20× diluted bacterial solution to monitor cell growth (Figure 3b).

### 3.3. Genetic Stability of the Recombinant Plasmid

In order to increase the expression of heterologous genes, recombinant strains carrying plasmids with heterologous genes were constructed. Antibiotics were needed to maintain the genetic stability of the plasmids during cultivation of recombinant strains and to remove them after fermentation, which increased the cost of production. However, antibiotic resistance genes are not allowed in the food industry. The food-grade recombinant strains based on the d-alanine racemase gene as a screening marker were constructed to make the product safer and lower cost. Here, the integrated recombinant *B.subitilis* D2/pMA5-*alrA*-*araA* was selected to conduct research. The plasmid showed better genetic stability when comparing the food-grade and the non-food-grade recombinant strains (Table 1). After 100 generations, the percentage of the recombinant plasmid containing the d-alanine racemase gene was above 90% without selection pressure, which indicated that the recombinant plasmid could easily be purified during subculture. 

### 3.4. Optimization of Whole-Cell Bioconversion

**Figure 4:**
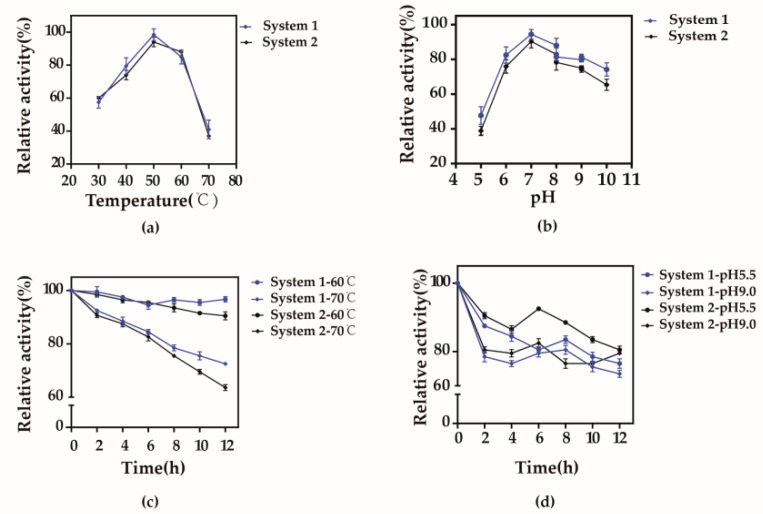
Optimum temperature and pH for whole-cell bioconversion. System 1: *B. subtilis* 168 D2/pMA5-*alrA*-*araA*; System 2: *B. subtilis* 168 D1/pMA5-*alrA*-*lacZ*-*araA*. Bioconversion measurements were performed at 0.1 mol/L Mn^2+^, 0.1% Triton X-100, and a cell concentration resulting in an OD of 40 at 600 nm. The temperature was set to a gradient from 30 to 70 °C (**a**). The following buffer systems were used to investigate the optimal pH: 0.1 M Na_2_HPO_4_-Citric buffer (pH 5–7), 0.1 M Tris-HCl buffer (pH 7–8), 0.1 M Glycine-NaOH buffer (pH 8–10). (**b**). To test the stability at relatively high temperatures, cells were exposed to 60 and 70 °C for 2–12 h during which conversion activity was measured (**c**). The pH stability of recombinant strains following the reaction was measured in pH 5.5 and 9.0 buffers for 2–12 h (**d**). Results are expressed as mean ± SD obtained from three independent experiments.

For both groups of the whole-cell bioconversion system, namely the integrated expression strains B. subtilis 168 D2/pMA5-alrA-araA and B. subtilis 168 D1/pMA5-alrA-lacZ-araA, the optimal temperature and pH were 50 °C and 7.0 (0.2 mol/L, citric acid—Na_2_HPO_4_), respectively (Figure 4a,b). Furthermore, the recombinant cells showed relatively good temperature stability, with 75% and 60% of the two strains still present after 12 h of incubation at a temperature of 70 °C (Figure 4c). The recombinant cells were relatively stable in acidic and alkaline buffers (Figure 4d).

**Figure 5:**
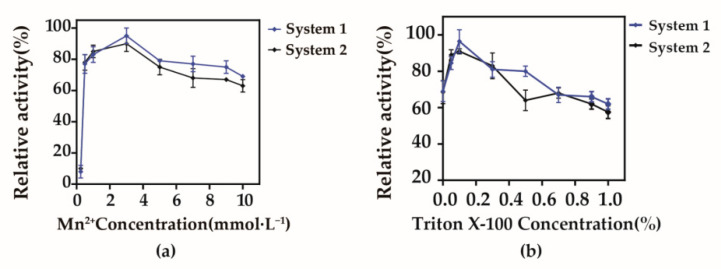
Optimum Mn^2+^ and TritonX-100 concentrations for whole-cell bioconversion. System 1: B. subtilis168 D2/pMA5-*alrA*-*araA*. System 2: *B. subtilis* 168 D1/pMA5-*alrA*-*lacZ*-*araA*. Bioconversion at 40 °C, pH 8 (0.2 mol/L citric acid-Na_2_HPO_4_), and cell concentration resulting in an OD of 40 at 600 nm. (**a**) Mn^2+^ concentration was set from 0 to 1 mol/L; (**b**) Triton X-100 concentration was set from 0 to 1%. Results are expressed as mean ± SD obtained from three independent experiments.

The metal ion Mn^2+^ can enhance the catalytic efficiency of arabinose isomerase to synthesize d-tagatose. The optimal concentration of Mn^2+^ was 3 mmol/L (Figure 5a). When the concentration of added Triton X-100 was 0.1%, the conversion rate was 1.2 times higher than with the original system (Figure 5b), and the time required to reach equilibrium was just 40 h (8 h less than the previous conversion time).

**Figure 6:**
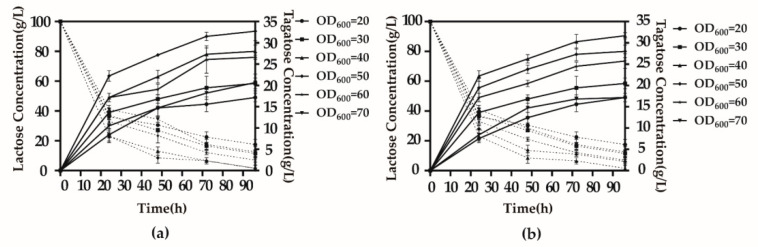
Optimum whole-cell bioconversion biomass. (**a**) *B.subtilis* 168 D2/pMA5-*alrA*-*araA*. (**b**) *B.subtilis* 168 D1/pMA5-*alrA*-*lacZ*-*araA*. Bioconversion was performed at 40 °C, pH 8 (0.2 mol/L citric acid-Na_2_HPO_4_), Mn^2+^ concentration of 0.1 mol/L, and 0.1% TritonX-100. Optimization of the concentration of whole cells occurred over the range of OD_600_ = 20–70. The solid line in the figure shows the real-time production of d-tagatose and the dashed line shows the real-time residual amount of the substrate lactose after the reaction. Results are expressed as mean ± SD obtained from three independent experiments.

The time required for the reaction to reach equilibrium was reduced when the concentration of the whole-cell suspension increased over a certain range, but at higher concentrations, the fluidity of the substrate and product decreased, and the conversion rate was reduced. The optimal concentration of cells (assessed by optical density [OD] at 600 nm wavelength) occurred at OD_600_ = 50 (Figure 6a,b).

### 3.5. d-tagatose Production

**Figure 7:**
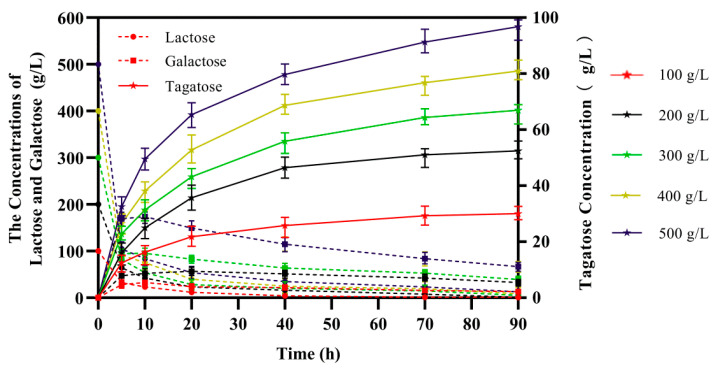
Biosynthesis of d-tagatose by the method of whole cell bioconversion. Solid lines and pentagram legends indicate immediate d-tagatose sugar production, dashed line and round legend shows the amount of the initial substrate lactose remaining in real-time, and the square legend shows its substrate galactose for arabinose isomerase. Reactions were carried out under the optimized conditions in a 1 L volume. The concentration of lactose ranged from 100 to 500 g/L. Results are expressed as mean ± SD obtained from three independent experiments.

Lactose was catalyzed into d-tagatose using the integrated recombinant cells *B. subtilis*168 D2/pMA5-*alrA*-*araA* under optimal catalytic conditions. *B. subtilis* cannot metabolize lactose but can metabolize glucose to acid but not gas, so the pH of the reaction system decreases during the reaction. Here, 4 M HCl and NaOH solutions were used to control pH during the process. The reaction reached equilibrium at 40 h and tended to be slow thereafter. The maximum molar conversion rate was 57.19%. The lactose concentration was set from 100–500 g/L, and the maximum yield of d-tagatose occurred when the substrate loading was increased to 500 g/L (Figure 7). For detailed HPLC results shown in Figure S1.

## 4. Discussion

In response to the serious worldwide problem of the increasing prevalence of diabetes and hyperglycemia, sweeteners are being developed and added to the daily diet to provide consumers with a calorie-free sweet taste. However, sugar cannot simply be replaced by intense sweetener, and d-tagatose is a highly productive and valuable sugar that is desirable for its low caloric content and ability to act as a bulk sweetener [15]. In both research and industrial production, *E. coli* is typically the host for construction of recombinant strains, but it has the disadvantage of producing enterotoxins. Likewise, antibiotics used to keep the plasmid stable increase the cost of downstream industrial processes. This study introduced a multi-copy plasmid expression system without additional antibiotics based on the CRE/*loxP* system and a food-safe *B. subtilis* strain as the expression host.

Lactose is common in dairy products and can be hydrolyzed by β-galactosidase [16]. It has a significant cost advantage over substrates, such as galactitol [1]. *E. coli*-derived β-galactosidase is potently hydrolytic, and its conversion of lactose produces large amounts of galactose that act as a reaction substrate for arabinose isomerase [5,7]. In this study, an attempt was made to construct two multi-enzyme expression systems consisting of β-galactosidase and arabinose isomerase. The CRE/*loxP* system can be utilized to delete specific gene fragments from a genome, and this feature was exploited to knock out alanine racemase in *B. subtilis*. d-alanine can be produced from l-alanine, and in the case of knocking out alanine racemase, cells grow on plates with added d-alanine. The alanine racemase gene *alrA* was ligated by homologous recombination to the plasmid pMA5, commonly used in *B. subtilis*, and then introduced into d-alanine-deficient *B. subtilis* by a chemical method. In this background, it was also possible to fuse a target gene at the position of *alrA* in the original genome, i.e., to integrate the expressed gene into the genome. This method has been used in an innovative way to safely produce d-tagatose [17,18]. The current study integrated the gene *lacZ*, encoding the key enzyme β-galactosidase, into the *B. subtilis* genome, while a constructed plasmid ligated with *alrA* that overexpressed arabinose isomerase was introduced. An initial expression system was constructed to balance the activities of both enzymes and achieve efficient transformation. For comparison and validation, the study overexpressed *lacZ* and *araA*, the gene encoding arabinose isomerase, on a pMA5 plasmid ligated with *alrA* to obtain a co-expression plasmid that was introduced into d-alanine-deficient *B. subtilis* to obtain a second expression system.

Compared with bioconversion using isolated enzymes in vitro, whole-cell bioconversion offers environmental benefits and lowers enzyme cost [4,19]. This study verified the optimum conditions for the production of d-tagatose by whole-cell bioconversion using the recombinant strains constructed here. The optimal pH of most reported arabinose isomerases varies over a range from neutral to alkaline [5]. Increasing the temperature shifts the equilibrium toward d-tagatose, but above a certain temperature, enzymes become unstable [20,21,22]. As is well known, Mn^2+^ significantly affects the activity of arabinose isomerase [5,23]. Because arabinose isomerase is negatively charged when phosphorylated, the decrease in pKa activates the enzyme’s active site, and the neutral sugar acidified by Mn^2+^ makes it easier to connect with the functional group on the enzyme. Therefore, Mn^2+^ can enhance the catalytic efficiency of the synthesis d-tagatose by arabinose isomerase. The overall efficiency of conversion is directly affected by movements of the substrate and product, which depend on the presence of specific transport proteins on the bacterial membrane. The structure or rigidity of the cell wall may be changed after treatment with permeabilizing agents, thereby achieving faster transport of the substrate and product [4,24]. Within a certain range, the time to reach equilibrium was reduced when the concentration of the whole-cell suspension was increased. However, when the cell concentration exceeded a certain range, the fluidity of the substrate and product decreased, and the conversion rate was reduced.

Finally, a 100–500 g/L concentration of the substrate lactose was used for transformation in a 1 L volume under optimal conditions. As the concentration of the lactose substrate increased, the conversion rate decreased to varying degrees because of the lower fluidity of the substrate and product. Conversely, an excessively high lactose concentration increased the transglycosidase activity of β-galactosidase, resulting in greater synthesis of galactooligosaccharides [8].

## 5. Conclusions

In this study, constructed recombinant *Bacillus subtilis* whole cells were utilized to transform cost-effective lactose to produce the promising sugar substitute d-tagatose, with a final yield of 96.8 g/L d-tagatose at 500 g/L of lactose and the highest molar conversion rate of 57.2%. Firstly, a heterologous gene expression system without the addition of additional antibiotics was constructed using *Bacillus subtilis* as the host. Subsequently, whole-cell transformation conditions were optimized to produce d-tagatose using recombinant strains. In summary, the above study can be applied to practical production to improve the d-tagatose yield and reduce costs.

## Figures and Tables

**Table 1:** Genetic stability evaluation of recombinant plasmids.

Generations	Stability of the Plasmid pMA5-*alrA*-*araA* (%)		Stability of the Plasmid pMA5-*araA* (%)	
	LB	LB (Add d-alanine)	LB	LB (Add d-alanine)
20	100 ± 0.00	98.00 ± 0.68	100 ± 0.00	96.00 ± 0.35
40	100 ± 0.00	96.43 ± 1.42	100 ± 0.00	94.60 ± 0.92
60	100 ± 0.00	95.00 ± 1.38	100 ± 0.00	93.48 ± 1.38
80	100 ± 0.00	93.27 ± 1.93	100 ± 0.00	92.02 ± 2.42
100	100 ± 0.00	92.30 ± 2.01	100 ± 0.00	90.54 ± 1.83

## Data Availability

HPLC spectroscopic data are available at the Key Laboratory of Industrial Biotechnology of Ministry of Education, School of Biotechnology, Jiangnan University.

## Supplementary Materials

The following are available online at https://www.mdpi.com/article/10.3390/biology10121343/s1, Figure S1: HPLC results of conversion products, Table S1: Comparison of characteristics of L-AIs from various microorganisms, Table S2. List of bacterial strains and plasmids used in this study.
