# Supplementary material for: Production of d-Tagatose by Whole-Cell Conversion of Recombinant Bacillus subtilis in the Absence of Antibiotics
Source: Biology (Basel). 2021 Dec 16;10(12):1343. doi: 10.3390/biology10121343 (PMC8698901; doi:10.3390/biology10121343)
Supplement: Supplementary file 1 [file biology-10-01343-s001.zip › biology-1503010-supplementary.pdf]

biology

MDPI

Supplementary Materials

Production of D-Tagatose by Whole-Cell Conversion of Recombinant *Bacillus subtilis* in the Absence of Antibiotics

Xian Zhang, Ruiqi Lu, Qiang Wang, Mengkai Hu, Zhiyue Li, Meijuan Xu, Taowei Yang, Rongzhen Zhang and Zhiming Rao

Table S1 . Comparison of characteristics of L-AIs from various microorganisms.

Source of L-arabinose isomerase	Reaction condition	Metal ion or boric acid requirement(mol/L)	con- cen- tra-	ose con- centra-	Conversion yield(%)
Mesophilic					
Lactobacillus sakei	pH7.4,50°C	Mn^{2+}（1.0）	54	45.9	85（48 h）
Lactobacillus brevis PC16	pH6.5,67°C	Mn^{2+}（1.0）	125	99.6	79.7(28 h)
Lactobacillus fermentum CGMCC 2921	pH6.5,65°C	Mn^{2+}（1.0）	9	4.95	55(12 h)
Lactobacillus fermentum CGMCC 2921	pH6.5,70°C	Mn^{2+}（1.0）	100	75	75(24 h)
Pediococcus pentosaceus PC-5	pH6.0,50°C	Mn^{2+}（0.6）Co2+(0.8)	5	2.6	52(24 h)
Enterococcus faecium DBFIQE36	pH5.5,50°C	Mn^{2+}（0.3）	90	40.5	45(48 h)
Lactobacillus plantarum WU14	pH7.17,50°C	Mn^{2+}（10.0）	108	41.4	38.31(28 h)
Bacillus coagulan NL01	pH7.5,60°C	Mn^{2+}（0.5）Co2+(0.5)	20	10.6	53(20 h)
Thermophilic					
Geobacillus therodenitrificans	pH8.5,50°C	No	300	165	55(2 h)
Geobacillus therodenitrificans	pH8.5-9.0, 60°C	boric acid (1110.0)	500	370	74(24 h)
Bacillus thermoglucosidasius KCTC 1828	pH7.4,40°C	Mn^{2+}（1.0）	36	16.42	45.6(48 h)
Acidothermus cellulolytic ATCC 43068	pH7.5,75°C	Mn^{2+}（1.0）Co^{2+}(0.5)	9	4.8	53(12 h)
Alicyclobacillus acidocaldarius	pH6.0,60°C	Mn^{2+}（1.0）Co^{2+}(1.0)	18	7.9	44(12 h)
Alicyclobacillus hesperidium URH17-3-68	pH7.0,70°C	Co^{2+}(1.0)	9	3.9	43(27 h)
Hyperthermophilic					
Thermotoga neapolitana 5068	pH7.0,80°C	Mn^{2+}（1.0）Co^{2+}(1.0)	1.8	1.2	68(20 h)
Thermotoga Maritima	pH7.5,70°C	Mn^{2+}（5.0）	500	190	38(242 h)

Host expressing system	Reference
Lactobacillus plantarum	[16]
B. subtilis DB403	[15]
E. coli	[14]
B. subtilis 168	[13]
E. coli	[12]
E. coli	[11]
Lactococcus lactis N79000	[10]
E. coli	[9]
Corynebacterium glutamicum	[8]
E. coli	[7]
E. coli	[6]
E. coli	[5]
E. coli	[4]
E. coli	[3]
E. coli	[2]
E. coli	[1]

Table S2. List of bacterial strains and plasmids used in this study.

Strains and plasmids	Characteristics	Source and reference
E. coli K-12	*β*-galactosidase and arabinose isomerase gene provider	Invitrogen
B. subtilis 168	trpC, expression host of *B. subtilis*	Takara
168 D1P1(*alrA*-, Zeo^r)	168 derivate, △*alrA*, lox71-Zeo^r-lox66	This study
168 D1P2(*alrA*-, Kan^r)	168 derivate, △*alrA*, lox72, Kan^r, pTSC	This study
168 D1(*alrA*-)	168 derivate, △*alrA*	This study
168 D1/pMA5-*alrA*	The recombinant 168 D1 strain harboring the plasmid pMA5-*alrA*	This study
168 D1/pMA5-*alrA-araA*	The recombinant 168 D1 strain harboring the plasmid pMA5-*alrA-araA*	This study
168 D1/pMA5-*alrA-lacZ*	The recombinant 168 D1 strain harboring the plasmid pMA5-*alrA-lacZ*	This study
168 D1/pMA5-*alrA-lacZ-araA*	The recombinant 168 D1 strain harboring the plasmid pMA5-*alrA-lacZ-araA*	This study
168 D2P1(Zeo^r)	168 derivate, *lacZ*, △*alrA*, lox71-Zeo^r-lox66	This study
168 D2P2(Kan^r)	168 derivate, *lacZ*, △*alrA*, lox72, Kan^r, pTSC	This study
168 D2(*alrA*-)	168 derivate, *lacZ*, △*alrA*	This study
168 D2/pMA5-*alrA*	The recombinant 168 D2 strain harboring the plasmid pMA5-*alrA*	This study
168 D2/pMA5-*alrA-araA*	The recombinant 168 D2 strain harboring the plasmid pMA5-*alrA-araA*	This study
**Plasmids**		
pMA5	Kan^r, Zeo^r, containing the *Hpa* II promoter	Takara
p7Z6	pMD18-T ligated with lox71-Zeo^r-lox66 cassette	Takara
pTSC	Kan^r, temperature sensitive in *B. subtilis* containing Pspac-cre expression cassette	Takara
pMA5-*alrA*	*B. subtilis* expression vector containing *alrA* gene	This study
pMA5-*alrA-araA*	*B. subtilis* expression vector containing *alrA* and *araA* genes	This study
pMA5-*alrA-lacZ*	*B. subtilis* expression vector containing *alrA* and *lacZ* genes	This study
pMA5-*araA*	*B. subtilis* expression vector containing *araA* gene	This study
pMA5-*alrA-lacZ-araA*	B. *subtilis* co-expression vector containing *alraA*, *araA* and *lacZ* gene	This study

Zeo^r: bleomycin resistance; Kan^r: kanamycin resistance;.

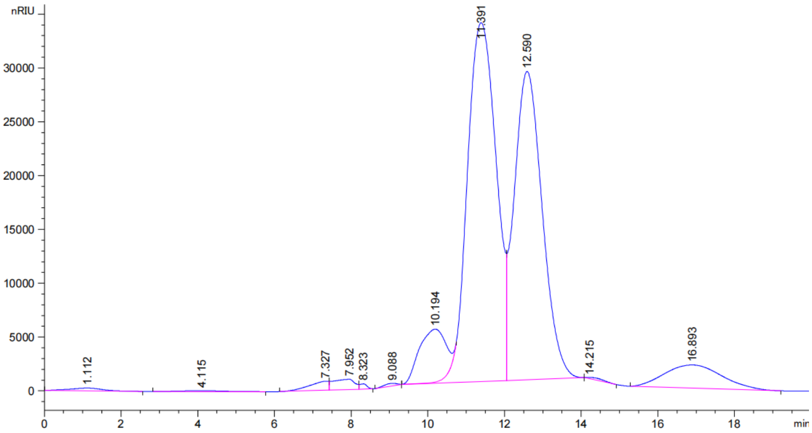

Figure S1. HPLC results of conversion products. The conversion using *B.subtilis* 168 D1/pMA5-*alrA-lacZ-araA* whole-cells. Bioconversion was performed at 40 °C, pH 8 (0.2 mol/L citric acid-Na_2HPO_4), Mn^{2+} concentration of 0.1 mol/L, and 0.1% TritonX-100, and cell concentration resulting in an OD of 40 at 600 nm. 10.194 min: lactose, 11.391 min: glucose, 12.590min: galactose, 16.893 min: D-tagatose.

References

1. Bortone, N.; Fidaleo, M. Stabilization of immobilized L-arabinose isomerase for the production of D-tagatose from D-galactose. *Biotechnol. Prog.* **2020**, *36*, e3033, doi:10.1002/btpr.3033.
2. Kim, B.C.; Lee, Y.H.; Lee, H.S.; Lee, D.W.; Choe, E.A.; Pyun, Y.R. Cloning, expression and characterization of L-arabinose isomerase from *Thermotoga neapolitana*: bioconversion of D-galactose to D-tagatose using the enzyme. *FEMS Microbiol. Lett.* **2002**, *212*, 121–126.
3. Fan, C.; Liu, K.; Zhang, T.; Zhou, L.; Xue, D.; Jiang, B.; Mu, W. Biochemical characterization of a thermostable L-arabinose isomerase from a thermoacidophilic bacterium, *Alicyclobacillus hesperidum* URH17-3-68. *Biotechnol. Prog.* **2003**, doi:10.1016/j.molcatb.2014.02.001.
4. Lee, S.J.; Lee, D.W.; Choe, E.A.; Hong, Y.H.; Kim, S.B.; Kim, B.C.; Pyun, Y.R. Characterization of a Thermoacidophilic L-Arabinose Isomerase from *Alicyclobacillus acidocaldarius*: Role of Lys-269 in pH Optimum. *Appl. Environ. Microbiol.* **2005**, *71*, 7888–7896, doi:10.1128/AEM.71.12.7888-7896.2005.
5. Cheng, L.; Mu, W.; Zhang, T.; Jiang, B. An L-arabinose isomerase from *Acidothermus cellulolytics* ATCC 43068: cloning, expression, purification, and characterization. *Appl. Microbiol. Biotechnol.* **2010**, *86*, 1089–1097, doi:10.1007/s00253-009-2322-z.
6. Seo, M.J. Characterization of an L-arabinose isomerase from *Bacillus thermoglucosidasius* for D-tagatose production. *Biosci. Biotechnol. Biochem.* **2013**, *77*, 385–388, doi:10.1271/bbb.120723.
7. Lim, B.C.; Kim, H.J.; Oh, D.K. High Production of d-Tagatose by the Addition of Boric Acid. *Biotechnol. Prog.* **2007**, *23*, 824–828.
8. Shin, K.C.; Sim, D.H.; Seo, M.J.; Oh, D.K. Increased Production of Food-Grade D-Tagatose from D-Galactose by Permeabilized and Immobilized Cells of *Corynebacterium glutamicum*, a GRAS Host, Expressing D-Galactose Isomerase from *Geobacillus thermodenitrificans*. *J. Agric. Food Chem.* **2016**, *64*, 8146–8153, doi:10.1021/acs.jafc.6b03588.
9. Mei, W.; Wang, L.; Zang, Y.; Zheng, Z.; Ouyang, J. Characterization of an L-arabinose isomerase from *Bacillus coagulans* NL01 and its application for D-tagatose production. *BMC Biotechnol.* **2016**, *16*, 55, doi:10.1186/s12896-016-0286-5.
10. Chang, X.; Ying, B.; Zhang, Y.; Cao, H.; Zhou, T.; Zhong, P.A.; Xu, B. The Study of Food-Grade Induced Expression and Enzymatic Properties of L-Arabinose Isomerase from *Lactobacillus plantarum WU*14 with High D-Tagatose Yield. *Food Nutr. Sci.* **2016**, *07*, 320–337, doi:10.4236/fns.2016.74034.
11. Manzo, R.M.; Antunes, A.; de Sousa Mendes, J.; Hissa, D.C.; Gonalves, L.R.B.; Mammarella, E.J. Biochemical Characterization of Heat-Tolerant Recombinant L-Arabinose Isomerase from Enterococcus faecium DBFIQ E36 Strain with Feasible Applications in D-Tagatose Production. *Mol. Biotechnol.* **2019**, *61*, 385–399, doi:10.1007/s12033-019-00161-x.
12. Men, Y.; Zhu, Y.; Zhang, L.; Kang, Z.; Izumori, K.; Sun, Y.; Ma, Y. Enzymatic conversion of D-galactose to D-tagatose: Cloning, overexpression and characterization of L-arabinose isomerase from *Pediococcus pentosaceus* PC-5. *Microbiol. Res.* **2014**, *169*, 171–178, doi:10.1016/j.micres.2013.07.001.
13. Liu, Y.; Li, S.; Xu, H.; Wu, L.; Xu, Z.; Liu, J.; Feng, X. Efficient production of D-tagatose using a food-grade surface display system. *J. Agric. Food Chem.* **2014**, *62*, 6756–6762, doi:10.1021/jf501937j.
14. Xu, Z.; Qing, Y.; Li, S.; Feng, X.; Xu, H.; Ouyang, P. A novel L-arabinose isomerase from *Lactobacillus fermentum* CGMCC2921 for D-tagatose production: Gene cloning, purification and characterization. *J. Mol. Catal. B Enzym.* **2011**, *70*, 1–7, doi:10.1016/j.molcatb.2011.01.010.

15. Guo, Q.; An, Y.; Yun, J.; Yang, M.; Magocha, T.A.; Zhu, J.; Xue, Y.; Qi, Y.; Hossain, Z.; Sun, W.; et al. Enhanced D-tagatose production by spore surface-displayed L-arabinose isomerase from isolated *Lactobacillus brevis* PC16 and biotransformation. *Bioresour. Technol.* **2018**, *247*, 940–946, doi:10.1016/j.biortech.2017.09.187.
16. Bober, J.R.; Nair, N.U. Galactose to tagatose isomerization at moderate temperatures with high conversion and productivity. *Nat. Commun.* **2019**, *10*, 4548, doi:10.1038/s41467-019-12497-8.
